# Exosome-delivered miR-153 from *Trichinella spiralis* promotes apoptosis of intestinal epithelial cells by downregulating Bcl2

**DOI:** 10.1186/s13567-023-01186-6

**Published:** 2023-06-28

**Authors:** Ruibiao Wang, Lihao Lin, Yang Han, Zhixin Li, Jingbo Zhen, Yuheng Zhang, Feng Sun, Yixin Lu

**Affiliations:** Heilongjiang Provincial Key Laboratory of Zoonosis, College of Veterinary Medicine, Northeast Agricultural University, Harbin, China

**Keywords:** *Trichinella spiralis*, exosomes, miR-153, Bcl2, apoptosis

## Abstract

Trichinellosis, a helminthic zoonosis, exhibits a cosmopolitan distribution and is a public health concern. In previous studies, it was reported that the exosomes secreted by *Trichinella spiralis* larvae (*Ts*Exos) largely affected cell biological activities. miRNAs, as exosome-delivered cargoes, affect the biological activities of the host by targeting genes. The present study aimed to elucidate the mechanisms by which miRNAs interact with intestinal epithelial cells. First, a miRNA library of *Ts*Exos was constructed; then, based on high-throughput miRNA sequencing results, miR-153 and its predicted target genes, namely, Agap2, Bcl2 and Pten, were selected for follow-up studies. The dual-luciferase reporter assays revealed that miR-153 directly targeted Bcl2 and Pten. Furthermore, real-time qPCR and Western blotting revealed that only Bcl2 was downregulated by *Ts*Exo-delivered miR-153 in porcine intestinal epithelial cells (IPEC-J2). Bcl2, an important antiapoptotic protein, plays an essential role in cell apoptosis as a common intersecting molecule of various signal transduction pathways. Therefore, we hypothesized that miR-153 derived from *Ts*Exos causes cell apoptosis by targeting Bcl2. The results suggested that miR-153 could induce apoptosis, reduce mitochondrial membrane potential, affect cell proliferation, and cause damage and substantial oxidative stress. Furthermore, miR-153 coincubated with IPEC-J2 cells stimulated the accumulation of the proapoptotic proteins Bax and Bad, which belong to the Bcl2 family of proteins, and the apoptosis-implementing proteins Caspase 9 and Caspase 3. Moreover, studies have suggested that miR-153 can promote apoptosis by regulating the MAPK and p53 signalling pathways involved in apoptosis. Thus, exosome-mediated miR-153 delivery secreted by *T. spiralis* could induce apoptosis and affect the MAPK and p53 signalling pathways by downregulating Bcl2 in IPEC-J2 cells. The study highlights the mechanisms underlying the invasion of *T. spiralis* larva.

**Supplementary Information:**

The online version contains supplementary material available at 10.1186/s13567-023-01186-6.

## Introduction

To date, parasitic diseases remain a growing concern worldwide, threatening human health [1]. Current studies mainly focus on the prosurvival mechanisms of parasites in the physiological or immunological environment of the host to achieve long-term parasitism via intercellular communication [2]. *Trichinella spiralis* is a foodborne zoonotic nematode with various mammalian hosts worldwide. *T. spiralis* infection is not only a major public hygiene problem but also poses a serious threat to meat safety [3, 4]. As a common intestinal parasite, *T. spiralis* releases specific proteins or other molecules that interfere with the immune response of host cells, evading detection and attack by the host immune system [5, 6]. Han et al. reported that *T. spiralis* excretory-secretory (ES) products may regulate host immune responses at the macrophage level [7]. In another study, as an indispensable component of the ES antigen of *T. spiralis*, a serine protease inhibitor could promote cell apoptosis by activating endoplasmic reticulum stress in intestinal epithelial cells [8]. Therefore, it is important to conduct additional functional studies on the secretory products of *T. spiralis* to understand the immune mechanisms of *T. spiralis*.

Recently, advances in exosomal research and its importance in cellular crosstalk, as well as the role played by exosomes in parasite invasion, have received increased attention [9-12]. Exosomes are membrane vesicles of endocytic origin that are released into the extracellular environment by different living cells [13]. As carriers, exosomes carry a series of bioactive cargos comprising miRNA, mRNA, DNA and proteins and transport them into cells for cell‒cell interplay and biological delivery [2]. The molecular composition of exosomes reflects the specialized functions of their origin. As they are transferred into target cells, exosomes probably modulate selected cellular activities, including vascular homeostasis and antigen presentation [14]. miRNAs are a class of noncoding single-stranded small RNAs with a length of 18–22 nt. They degrade mRNA by binding to the 3′ untranslated region (3′-UTR) of target mRNAs, leading to inhibition of mRNA translation [15].

In a previous study, the effects of exosomes derived from *T. spiralis* on the barrier function of small intestinal epithelial cells were explored. These exosomes were found to be involved in several cellular biological processes, which ultimately compromised cellular barrier function [16]. Building on the results of a previous study and the nature of exosomes as delivery vehicles, we hypothesized that the biologically active substances delivered by exosomes played a functional role in the entire process. Therefore, in the present study, the focus was on exosomal miRNAs as cargoes to investigate the effects of the miRNAs of *T. spiralis* exosomes (*Ts*Exos) on intestinal epithelial cells.

## Materials and methods

### Animals and parasite

Female KM mice (25–30 g) were purchased from Harbin Medical University. All mice were maintained under specific pathogen-free (SPF) conditions according to the Chinese Animal Management Ordinance. The study was conducted in accordance with the recommendations of and was approved by the Animal Management Committee of the Northeast Agricultural University.

*T. spiralis* (strain ISS533) was cultured in KM mice, and muscle larvae (ML) were isolated from the muscles of infected mice using the standard method described previously [17].

### Establishment of an exosomal miRNA library

*T. spiralis* ML were collected from the muscles of mice on the 40^th^ day post-infection and cultured in RPMI-1640 medium with 1% 100 U/mL penicillin/streptomycin at 37 ℃ under 5% CO_2_ for 48 h. The exosomes secreted in the culture supernatant were isolated by the method described by Lässer et al. [18]. The results of *Ts*Exo identification were found in a previous article [16]. Each time before proceeding with downstream experiments, *Ts*Exos were subjected to transmission electron microscopy (TEM) (Hitachi, Japan) to confirm their quality, and the concentration of *Ts*Exos was measured using a BCA protein assay kit (Meilunbio, China).

Total RNA was extracted from the exosomes and used for high-throughput miRNA sequencing. Library preparation and miRNA sequencing were performed by Bioacme (Wuhan, China). Briefly, total RNA was used as the experimental material for PCR to obtain a small RNA (sRNA) library. The qualified sRNA library was subjected to onboard sequencing using the Illumina HiSeq™ 3000 platform (Illumina, USA). Clean sequencing reads were obtained using the related analysis software of Illumina.

### Quantitative real-time PCR

Relative miRNA expression was evaluated using quantitative real-time polymerase chain reaction (qPCR). The forward primer sequences for each detected miRNA were obtained from the established miRNA library (Table 1), and the universal PCR reverse primer was purchased from Sangon Biotech (China). Total miRNA was extracted from the exosomes using the Total RNA Extraction kit (Solarbio, China). After synthesis of cDNA from total RNA using miRNA first-strand cDNA synthesis (tailing reaction), qPCRs were performed using the Roche Light Cycler 480 system. The results were calculated according to the method described by Livak et al. [19]. The qPCRs in subsequent cell assays were performed according to the method as a reference, and the primers used for subsequent qPCR are presented in Table 2.

**Table 1:** **Primers of detected miRNAs for qPCR.**

miRNA	Forward primers
miR-1	TGGAATGTAAAGAAGTATGTA
miR-9	TCTTTGGTTATCTAGCTGTATGA
miR-86	TAAGTGAATGCTTTGCCACAGACT
miR-100	AACCCGTAGATCCGAACTTGTGTT
miR-153	TTGCATAGTCACAAAAGTGA
miR-228	AATGGCACTGGATGAATTCACG
miR-252	CTAAGTAGTAGTGCCGCAGGTC
miR-263	CTTGGCACTGTAAGAATTCACAGA
Novel-miR-4	TGAGATCACCGTGAAAGCCTTTA
Novel-miR-8	AATGGCACTGGATGAATTCACA
Novel-miR-14	TAAGTGAATGCTTTGCCACAGACC
Novel-miR-25	TGGACGGATGCTCAGTGGATG
Novel-miR-31	CTACGATCATCTTTGCTCAATT
Novel-miR-32	TTGAGCAATCACAGTCGTA
Novel-miR-42	TCTTTGGTCATTTAGCTGTATGA
Novel-miR-46	TAAAAGACTGTGTGACTTCTACT
U6	CGCTTCGGCAGCACATATAC

**Table 2:** **Primers of the detected genes for qPCR.**

Gene name	Primers	Primer sequence (5′–3′)	Accession Number
Bcl-xL	Forward	5′-GTGCGTGGAGAGCGTAGACAAG-3′	NM_214285.1
	Reverse	5′-ACCATCGGTTGAAGCGTTCCTG-3′	
Bax	Forward	5′-ATCTACCAAGAAGTTGAGCGAGTGT-3′	XM_003127290.5
	Reverse	5′-CCAGTTGAAGTTGCCGTCAGC-3′	
BAD	Forward	5′-ACCGAGGAGGATGAAGGGACTGA-3′	XM_021082883.1
	Reverse	5′-GGAACCCTGGAACTCGTCACTCA-3′	
BID	Forward	5′-CCGCACAGTTCAGGAACCAGAG-3′	NM_001030535.1
	Reverse	5′-GCCAGGAGCATAGTCAGCACAAG-3′	
Caspase 9	Forward	5′-CCTTCCTGTGTTCATCTCCTGCTTA-3′	XM_003127618.4
	Reverse	5′-TGGCTCCTCTGGCTTGAGTTCC-3′	
Caspase 3	Forward	5′-GCCGAGGCACAGAATTGGACT-3′	NM_214131.1
	Reverse	5′-TTCGCCAGGAATAGTAACCAGGTG-3′	
ERK1	Forward	5′-ACGACCACATCTGCTACTTCCTCT-3′	XM_021088019.1
	Reverse	5′-CCACATATTCCGTCAAGAAGCCAGT-3′	
ERK2	Forward	5′-CAGCACCTCAGCAACGACCATATC-3′	NM_001198922.1
	Reverse	5′-GTGTTGAGCAGCAGGTTGGAAGG-3′	
MEK1	Forward	5′-TGGAGGTGTGGTGTTCAAGGTCT-3′	NM_001143716.1
	Reverse	5′-ATCTCGCCATCGCTGTAGAATGC-3′	
p38	Forward	5′-ACGAGACCTCCGCCTGTGAA-3′	NM_001243673.1
	Reverse	5′-TCCTCTTCCTGTCCTCCACCTTC-3′	
p53	Forward	5′-ACCGCCGCACAGAGGAAGAA-3′	NM_213824.3
	Reverse	5′-CGTCATTCAGCTCTCGGAACATCTC-3′	
PI3K	Forward	5′-ACAGCAAGCAGCACGAGTAACC-3′	NM_001244503.1
	Reverse	5′-GCCTGGACTCCGACTTCTGGTAA-3′	
AKT	Forward	5′-CGGCAGGAGGAAGAGATGATGGA-3′	NM_001159776.1
	Reverse	5′-AGCAGCTTCAGGTACTCGAACTCA-3′	
GAPDH	Forward	5′-GGTGAAGGTCGGAGTGAACG-3′	NM_001206359.1
	Reverse	5′-CCGTGGGTGGAATCATACTGG-3′	

### Cell culture, growth and transfection

Porcine small intestinal epithelial cells (IPEC-J2) and human embryonic kidney 293T cells (293T) were donated by Harbin Veterinary Research Institute. These cells were cultured in 90% DMEM with 10% foetal bovine serum (FBS) (PAN, Germany) and 1% 100 U/mL penicillin/streptomycin. Cells were transfected using Lip2000 (JTS, China) following the manufacturer’s instructions. For the dual-luciferase assay, miR-153 mimics/inhibitor (sequences are presented in Table 3; synthesized by GenePharma, China), plasmid, and Lip2000 were diluted with Opti-MEM (Gibco, USA). Note: miRNA mimics refer to synthetic RNA molecules designed to mimic the function of miRNAs; miRNA inhibitors refer to synthetic oligonucleotide molecules designed to inhibit the activity of miRNAs. After incubation, the transfection solution was cocultured with 293T cells. After 6 h, fresh medium was added, and the transfected cells were fed and grown for an additional 24 h. For the IPEC-J2 cell assays, the miRNA mimics or inhibitor were transfected into cells using Lip2000. After 6 h, the transfected cells were fed fresh medium or cocultured with *Ts*Exos and then grown for an additional 24 h. The reference concentration of *Ts*Exos was obtained from a previous study [16].

**Table 3:** **Sequencing of miRNA mimics and inhibitor.**

miRNA	Sequence
miR-153 mimics	5′-UUGCAUAGUCACAAAAGUGA-3′
	5′-UCACUUUUGUGACUAUGCAA-3′
miR-153 mimics NC	5′-CUCCGAACGUGUCACGUTT-3′
	5′-GUGACACGUUCGGAGAATT-3′
miR-153 inhibitor	5′-UCACUUUUGUGACUAUGCAA-3′
miR-153 inhibitor NC	5′-GUACUUUUGUGUAGUACAA-3′

### Screening of potential miRNAs

First, qPCR was performed to verify the *Ts*Exo-packaged miRNAs. In brief, different treatment groups were prepared: *Ts*Exos were incubated with 100 µg/mL RNase A (RNA digestion reagent; Beyotime, China) at 37 °C for 2 h; *Ts*Exos were coincubated with RNase A and 1 mg/mL Proteinase K (Protein degradation reagent; Beyotime, China) at 37 °C for 2 h; after treatment with RNase A and Proteinase K, *Ts*Exos were incubated with 0.25% Triton X-100 (used to disrupt the membrane; Biofroxx, Germany) at room temperature for 10 min. Moreover, a PBS control group was set up. Following treatment completion, miRNAs were detected via qPCR. Second, based on the results of qPCR and high-throughput miRNA sequencing, an assay was performed to validate whether the screened candidate miRNAs packaged in the exosomes could be transferred into IPEC-J2 cells. For this, *Ts*Exos were cocultured with IPEC-J2 cells at different time points. After incubation, total RNA was harvested from the cells, and candidate miRNA and pre-miRNA levels were measured by qPCR. The forward primer for the candidate pre-miRNA was as follows: AGCGGTGGCCAGTGTCATTT, and a common antisense primer was obtained from Sangon Biotech.

### Vector constructions

The pmirGLO Dual-luciferase vectors (MiaoLingbio, China) were used to construct the recombinant plasmids. The PCR-amplified fragment of the mRNA 3′-UTR obtained from IPEC-J2 cells was cloned into the pMD18-T vector (TaKaRa, Japan) and then inserted into pmirGLO to construct the recombinant plasmid pmirGLO-WT (wild type). The primers for target genes are presented in Table 4. Then, according to the binding sites of the miRNA‒mRNA predicted using the RNAhybrid online website, the mutant sequences of target genes were designed, and the recombinant plasmid pmirGLO-MUT (mutative type) was synthesized by General Biol (China).

**Table 4:** **The primers of miRNA‒target mRNA 3′-UTR.**

Genes	Primers	Sequence	Gene_ID
Agap2	Forward	5′-CCGCTCGAGGCGTTTCAGGGTAGGGGAAGCCAAG-3′	XM_021091890.1
	Reverse	5′-ACGCGTCGACTCACGGGGTTGAGGGATGGGTCTGG-3′	
Bcl2	Forward	5′-CCGCTCGAGTGGAGCGTGAACCTGGGAGCTAAGG-3′	XM_021099593.1
	Reverse	5′-ACGCGTCGACTGTCATTGTAGTTTGGTCTC-3′	
Pten	Forward	5′-CCGCTCGAGTAAACTGAAAATGGACCTTTTTTTT-3′	NM_001143696.1
	Reverse	5′-ACGCGTCGACTATTTATCCTAATTGAATTTTAAA-3′	

The protective bases are underlined in red, and the enzyme loci are underlined in green.

### Dual-luciferase reporter gene assay

After transfection, a dual-luciferase reporter gene assay kit (Yeasen, China) was used to detect firefly luciferase activity. Both firefly and Renilla luciferase activities were measured using a microplate reader (Tecan, Switzerland). Luciferase activity was calculated by normalizing the firefly luciferase activity relative to the Renilla luciferase activity. The different transfection groups of 293T cells are shown in Table 5.

**Table 5:** **Dual-luciferase assay groups (miRNA-Bcl2 mRNA target pairs).**

Groups	Components
Group 1	Control (untransfected cells)
Group 2	pmirGLO + miR-153 mimics
Group 3	pmirGLO + miR-153 mimics NC
Group 4	pmirGLO + miR-153 inhibitor
Group 5	pmirGLO + miR-153 inhibitor NC
Group 6	pmirGLO-Bcl2 3′UTR-WT + miR-153 mimics
Group 7	pmirGLO-Bcl2 3′UTR-WT + miR-153 mimics NC
Group 8	pmirGLO-Bcl2 3′UTR-WT + miR-153 inhibitor
Group 9	pmirGLO-Bcl2 3′UTR-WT + miR-153 inhibitor NC
Group 10	pmirGLO-Bcl2 3′UTR-Mut + miR-153 mimics
Group 11	pmirGLO-Bcl2 3′UTR-Mut + miR-153 mimics NC
Group 12	pmirGLO-Bcl2 3′UTR-Mut + miR-153 inhibitor
Group 13	pmirGLO-Bcl2 3′UTR-Mut + miR-153 inhibitor NC

Dual-luciferase assay groups of miRNA-Agap2 or Pten mRNA target pairs refer to the group of Bcl2.

### Western blotting

Total proteins were extracted from the cells, and the protein concentration was determined using a BCA assay kit. The proteins were fractionated by using 12% sodium dodecyl sulfate/polyacrylamide gel electrophoresis (SDS‒PAGE) and transferred onto a polyvinylidene fluoride filter membrane (PVDF; Millipore, USA). After incubation with primary antibodies (Wanleibio, China; Abclonal, USA), the PVDF membrane was washed and incubated with horseradish peroxidase (HRP)-conjugated secondary antibody (Abclonal). Finally, the blot was developed using ultrasensitive ECL chemiluminescence reagent (Meilunbio), and the membrane was exposed. The bands were quantified using a chemiluminescence imaging system (Syngene, USA) and analysed using ImageJ.

### Cell viability, permeability, cytotoxicity, and oxidative stress assays

After the treatments, cell viability, cytotoxicity, and oxidative stress assays were performed using a CCK-8 kit, lactate dehydrogenase (LDH) assay kit, and reactive oxygen species (ROS) assay kit (Meilunbio), respectively. The assays were performed according to the manufacturers’ instructions. The permeability of the monolayer IPEC-J2 cells was measured by performing the FITC-dextran assay (40 kDa; Yuanyebio, China) described previously [16].

### Cell apoptosis assay

The miRNA-transfected and *Ts*Exo-cultured cells were detected using the Annexin V-FITC/PI Cell Apoptosis Kit (Meilunbio) via flow cytometry (FCM). Cells that were stained with PI and FITC were identified as apoptotic cells. Hoechst 33258 (Leagene, China) staining was performed to confirm apoptosis by observing the intensity of blue fluorescence. Mitochondrial membrane potential was measured using a JC-1 Mitochondrial Membrane Potential Assay Kit (Solarbio) under a fluorescence microscope (Syngene).

### Statistical analysis

SPSS 22, GraphPad Prism 5, and other software programs were used for statistical analysis. All data are expressed as the mean ± SD. Differences between the groups were assessed using one-way analysis of variance (ANOVA) and Dunnett’s post hoc test. A *P-*valve of < 0.05 was considered statistically significant. The quantification of the protein band intensities was analysed using ImageJ software.

## Results

### Exosomal miRNA library

**Figure 1:**
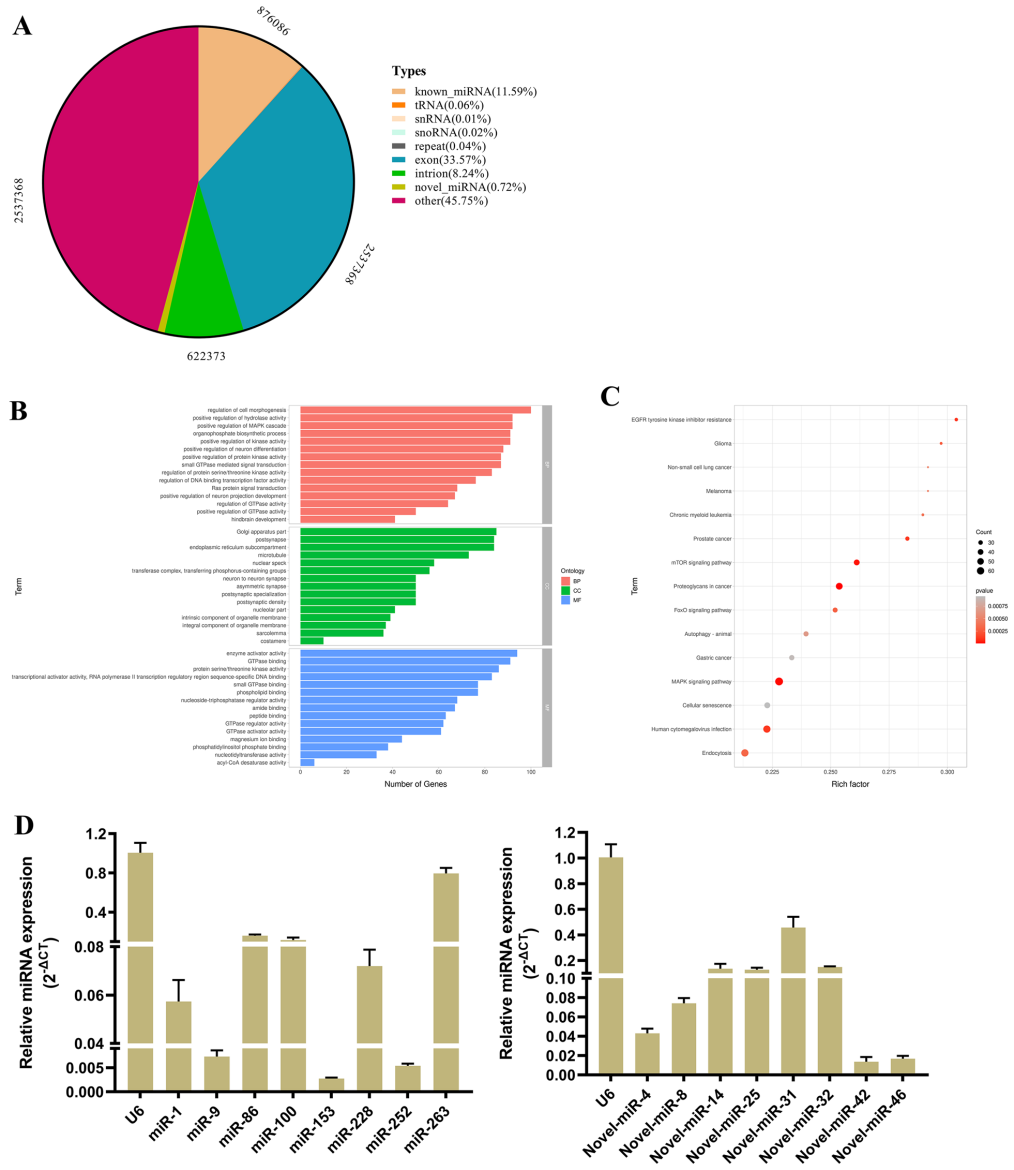
**
miRNA library of*Ts*Exos. A** Statistical graphs of small RNAs (sRNAs). **B** Box plot of Gene Ontology (GO) enrichment. BP: biological process; CC: cellular component; MF: molecular function. **C** Dot plot of KEGG enrichment. **D** Determination of miRNA levels by qPCR.

The exosomal miRNAs derived from the muscle larvae of *T. spiralis* were subjected to high-throughput sequencing. The miRNA library contained 876 086 known miRNA reads, accounting for 11.59% of small RNAs (sRNA) and originating from 48 miRNA families, and 54,227 unknown miRNA reads, accounting for 0.72% of sRNA, with 76 novel miRNAs (Figure 1A, and Tables 6 and 7). To predict the target mRNAs for the known and novel miRNAs, we selected the 3′-UTR of *Sus scrofa* mRNA in the target gene prediction database. Furthermore, PITA, RNA22, miRmap, microT, miRanda, PicTar, and TargetScan software or online tools were used for target gene prediction. The target mRNAs were subjected to Gene Ontology (GO) and Kyoto Encyclopedia of Genes and Genomes (KEGG) enrichment analyses, with the significance threshold set at *P* < 0.05. GO and KEGG enrichment analyses are presented in Figures 1B and C.

**Table 6:** **The known miRNA sequences in** ***Ts*Exos (Top 15).**

miRNA	Reads	Mature miRNA in the arm	Sequences with the highest abundance
miR-263	553804	5′	cuuggcacuguaagaauucacaga
miR-86	114,06	5′	uaagugaaugcuuugccacagAcu
miR-100	49583	5′	aacccguagauccgaacuuguguu
miR-228	45772	5′	aauggcacugGaugaauucacg
miR-1	37314	3′	uggaauguaaagaaguaugua
miR-9	21007	5′	ucuuugguuaucuagcuguauga
miR-252	11623	5′	cuaaguaguagugccgcagguC
miR-153	11357	3′	uugcauagucacaaaagugaug
let-7	7970	5′	ugagguaguagguuguauaguu
miR-57	5851	5′	uacccuguagCaccgagcugugu
miR-87	2716	3′	gugagcaaaguuucaggugugu
miR-125	2016	5′	ucccugagacccAaacuugugaa
miR-29	1501	3′	uagcaccauuugaauucagu
miR-133	1327	3′	auugguccccuucaaccagcu
miR-72	1072	5′	aggcaagauguuggcauagcuga

5′-or-3′ arm: The location of mature miRNA in precursor miRNAs; the capitalized bases indicate base differences from the reference sequence.

**Table 7:** **
Unknown miRNA sequences in** ***Ts*Exos (top 15).**

Novel-miR	Reads	Mature miRNA in the arm	Reference sequences
Novel-miR-31	9841	5′-and-3′	cuacgaucaucuuugcucaauu
Novel-miR-32	6346	3′	uugagcaaucacagucgua
Novel-miR-25	3502	3′	uggacggaugcucaguggaug
Novel-miR-14	3239	5′-and-3′	uaagugaaugcuuugccacagacC
Novel-miR-8	2516	5′	aauggcacuggaugaauucacA
Novel-miR-4	2034	3′	ugagaucaccgugaaagccuuuA
Novel-miR-46	1982	3′	uaaaagacugugugacuucuacu
Novel-miR-42	1834	5′	ucuuuggucauuuagcuguauga
Novel-miR-28	1707	5′	uacccuguagcaccgagcugugugu
Novel-miR-29	1671	3′	uacccguaucucuucuugguuc
Novel-miR-33	1534	3′	uugagcaacucugguugucgcuA
Novel-miR-37	1403	5′	uacgacugugauugcucaauug
Novel-miR-55	1349	5′-and-3′	uucugugcuguauccgauGaau
Novel-miR-30	1134	5′-and-3′	aacccguauuuuccucuuagucu
Novel-miR-61	781	5′	ucaccggauacuaaaacacgugu

5′-or-3′ arm: The location of mature miRNA in precursor miRNAs; the capitalized bases indicate base differences from the reference sequence.

To validate the miRNA sequencing results, we selected the top eight known miRNAs and top eight novel miRNAs with the highest abundance for qPCR analysis. U6 was used as a reference gene for miRNA identification. The qPCR results revealed that the transcriptional levels of the known and novel miRNAs were consistent with the sequencing results, further validating the accuracy of sequencing (Figure 1D).

### Determination of miRNAs and target genes

**Figure 2:**
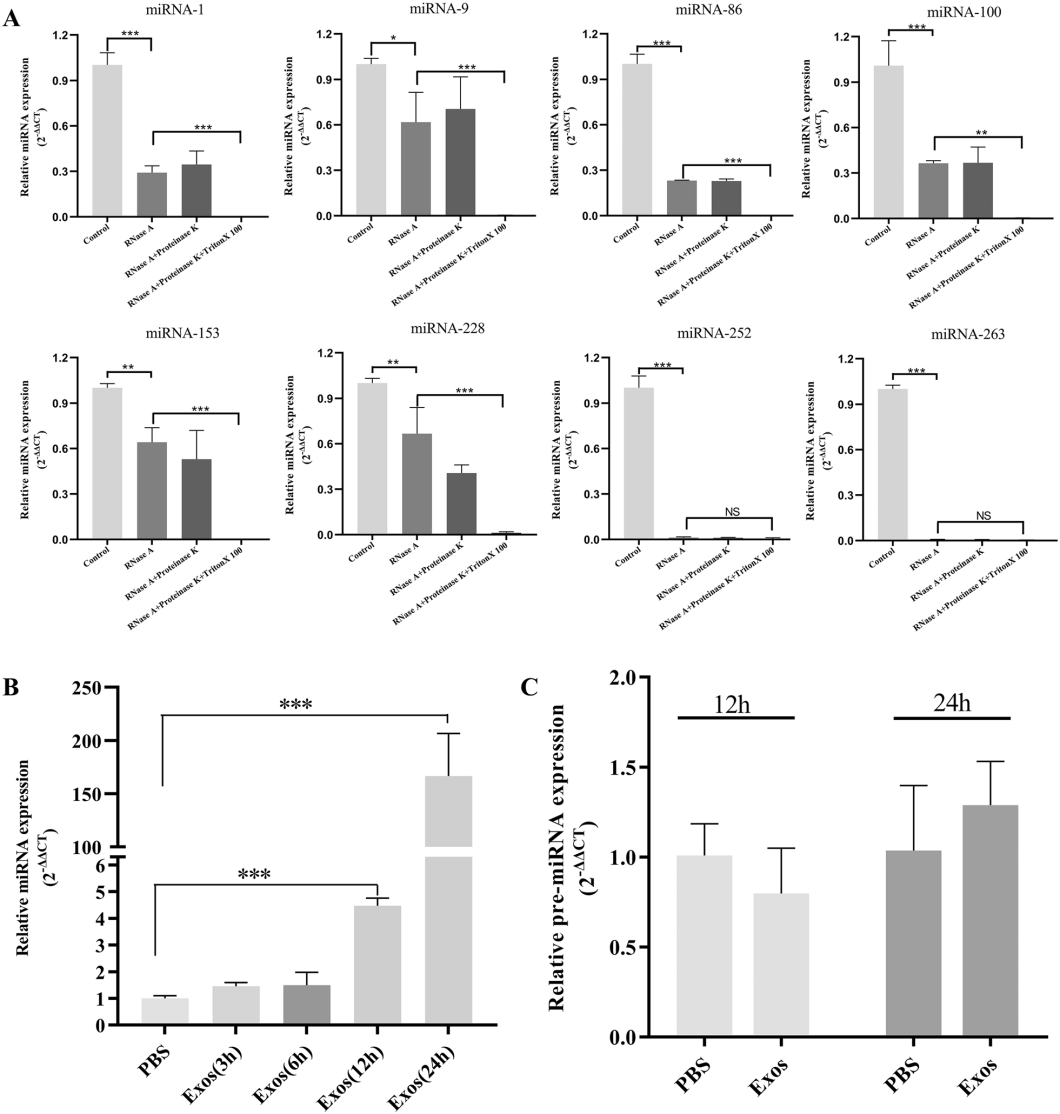
**
Determination of candidate miRNAs.** **A** Identification of the most abundant miRNAs within *Ts*Exos by qPCR. **B ***Ts*Exos were incubated with IPEC-J2 cells for 3, 6, 12, and 24 h to evaluate the miR-153 content in cells by qPCR. **C** The *Ts*Exos were incubated with IPEC-J2 for 12 and 24 h to evaluate the pre-miR-153 content in cells by qPCR. The data were analysed from 3 independent experiments, and the data are expressed as the mean ± SD. *P < 0.05, **P < 0.01, ***P < 0.001 compared with the PBS group.

Due to the limited quantity of lipoproteins and other substances in the exosomes by ultracentrifugation, according to the miRNA sequencing results, we further verified whether the top eight abundant miRNAs were packaged in *Ts*Exos. The results showed that after treatment with RNase A, proteinase K, and Triton X-100, the levels of miR-1, 9, 86, 100, 153, and 228 were significantly decreased compared to those with treatment with RNase A alone (*P* < 0.001), indicating that these miRNAs were mainly packaged in the exosomes, whereas miR-252 and 283 were mainly present outside (Figure 2A). Combining miRNA abundance data, KEGG/GO enrichment analysis results, the predicted miRNA target genes, and the results of miRNAs within the exosomes, we finally selected miR-153 and its predicted target genes Agap2, Bcl2, and Pten for subsequent studies (Tables 8 and 9). Furthermore, confirmation was conducted to determine whether miR-153 was carried into the IPEC-J2 cells by *Ts*Exos. The results suggested that there was no significant difference in miR-153 levels at the 3- and 6-h time points (*P* > 0.05); however, the miR-153 level was significantly increased at the 12- and 24-h time points compared with the control (*P* < 0.001); the level continuously increased as time increased (Figure 2B). As shown in Figure 2C, after coincubation of IPEC-J2 cells with exosomes for 12 or 24 h, no significant difference in pre-miR-153 content was observed compared with the control (*P* > 0.05); this indicated that the increase in miR-153 content in IPEC-J2 cells was mainly derived from *Ts*Exos rather than from the activation of the endogenous miR-153 synthesis system.

**Table 8:** **Partial KEGG enrichment pathways in the miRNA library.**

No._KEGG	Signal pathway	No._Target genes
ssc04010	MAPK	67
ssc04151	PI3K-Akt	67
ssc04014	Ras	47
ssc04024	cAMP	46
ssc04150	mTOR	41
ssc04140	Autophagy-animal	34
ssc04068	FoxO	33
ssc04210	Apoptosis	27
ssc04152	AMPK	26
ssc04350	TGF-beta	20
ssc04115	p53	17
ssc04215	Apoptosis—multiple species	11

**Table 9:** **The miR-153 predicted target genes and pathways.**

Gene_ID	Gene_Name	KEGG pathways
XM_021098320.1	Ide	Alzheimer disease
NM_001097442.1	Dab1	Spinocerebellar ataxia
NM_001143696.1	Pten	Inositol phosphate metabolism/EGFR tyrosine kinase inhibitor resistance/FoxO signalling pathway/p53 signalling pathway/Autophagy–animal/mTOR signalling pathway/PI3K-Akt signalling pathway/PD-L1 expression and PD-1 checkpoint pathway in cancer/Focal adhesion
NM_001101816.2	p35	Alzheimer disease/Pathways of neurodegeneration-multiple diseases/Cocaine addiction
XM_021091890.1	Agap2	FoxO signalling pathway/Endocytosis
XM_021078159.1	Sgk2	FoxO signalling pathway/PI3K-Akt signalling pathway
XM_021099593.1	Bcl2	EGFR tyrosine kinase inhibitor resistance/NF-kappa B signalling pathway/p53 signalling pathway/Autophagy – animal/PI3K-Akt signalling pathway/Apoptosis/NOD-like receptor signalling pathway/JAK-STAT signalling pathway
NM_001031787.1	Rab1a	Autophagy–animal/Amyotrophic lateral sclerosis/Pathways of neurodegeneration-multiple diseases/Legionellosis

### Identification of the targeting relationship between miRNA and mRNA

**Figure 3:**
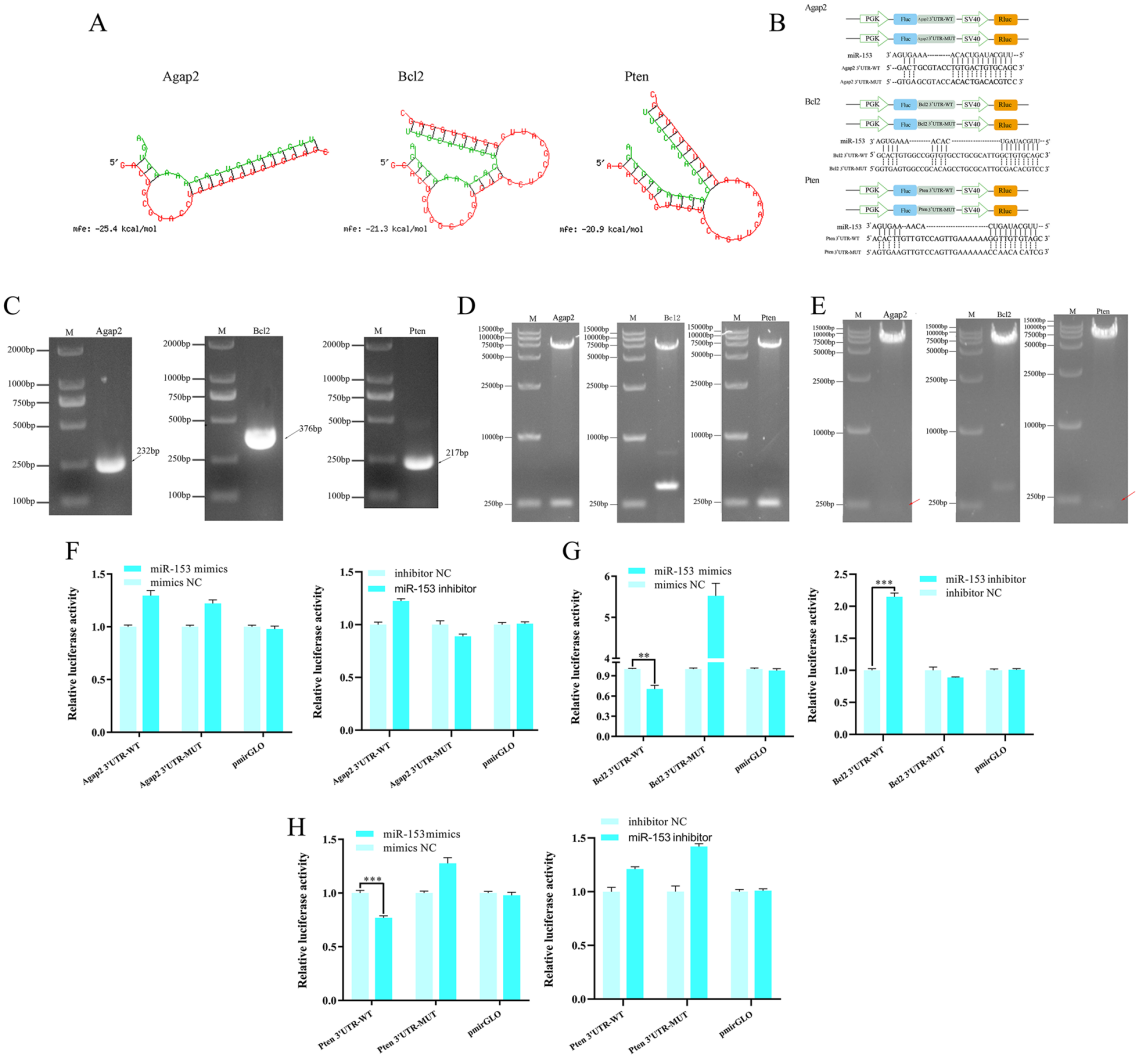
**
Verification of the targeting relationship between miR-153 and Agap2, Bcl2 and Pten. A** Base pairings between the mRNA targets (Agap2, Bcl2 and Pten) and miR-153 were predicted by RNAhybrid. **B** Schematic representation of point mutations. Promoter: PGK, SV40; Fluc: firefly luciferase; Rluc: Renilla luciferase. The electrophoresis results of (**C**) PCR amplification of the target gene fragment (Agap2 232 bp, Bcl2 376 bp, and Pten 217 bp), **D** double-enzyme digestion of recombinant pmirGLO-WT vectors, and **E** double-enzyme digestion of recombinant pmirGLO-MUT vectors. The miR-153 and Agap2 **F**, Bcl2 **G** and Pten **H** mRNA targeting relationships were detected by a dual-luciferase assay system. Calculation formula of the dual-luciferase assay: Ratio = F (experimental group - control group)/R (experimental group–control group). F: Firefly luciferase activity; R: Renilla luciferase activity; The control group: untransfected cells. *P < 0.05, **P < 0.01, and ***P < 0.001 indicate the significance levels of the comparisons between the mimic NC group and the mimic group, as well as between the inhibitor NC group and the inhibitor group.

The mRNA 3′-UTR sequences of the target genes Agap2, Bcl2, and Pten were obtained from GenBank. The binding sites between miR-153 and the 3′-UTR of target genes were predicted using the online tool RNAhybrid, and the minimum free energy value should be −20 kcal/mol. Based on the binding sites of miR-153 to the 3′-UTRs of Agap2, Bc12, and Pten mRNA obtained from RNAhybrid (Figure 3A), primers were designed to amplify the fragments containing the binding sites. With total DNA from IPEC-J2 cells as the template, PCR amplification successfully resulted in the production of Agap2 (232 bp), Bcl2 (376 bp), and Pten (217 bp) fragments (Figure 3B); these three gene sequences had an identity of 100% with the nucleotide sequences registered in GenBank. Then, three different recombinant plasmids were constructed by inserting the three target fragments into the 3′-UTR of the firefly luciferase gene in the pmirGLO vector. The recombinant plasmids were identified via double digestion with XhoI and SalI (TaKaRa, Japan). In subsequent electrophoresis, a 7350 bp pmirGLO band and the corresponding band for the exogenous genes were obtained (Figure 3D), and the results of sequencing confirmed a 100% sequence concordance (Additional file 1), indicating the successful construction of three kinds of recombinant plasmids: pmirGLO-Agap2/Bcl2/Pten 3′-UTR-WT. Likewise, according to the potential miRNA targeting sites within the 3′-UTRs, point mutations were introduced by a single base substitution (A → T, T → A, G → C, and C → G). The schematic representation of gene point mutations is shown in Figure 3C, and the mutant sequences are presented in Additional file 2. Immediately after, three kinds of mutant 3′-UTRs were inserted into pmirGLO plasmids. Figure 3E and Additional file 3 demonstrate that three different pmirGLO-MUT plasmids were also obtained after double digestion verification and sequencing comparison.

The 293T cells were transfected with the pmirGLO luciferase construct (pmirGLO, pmirGLO-MUT or pmirGLO-WT) and miR-153 mimics or inhibitor, and the dual-luciferase reporter gene assay was performed to verify the targeting associations of miRNA‒mRNA. In the verification assays for Bcl2 and Pten mRNA, after normalization of the relative firefly luciferase activities against Renilla luciferase activities, the luciferase activity assay showed that miR-153 markedly inhibited firefly luciferase activity (*P* < 0.01; *P* < 0.001) (Figures 3G and H); however, in the assay for Agap2 mRNA, there were no alterations in firefly luciferase activity in Figure 3F. These results indicated that miR-153 could directly target Bcl2 and Pten mRNAs rather than Agap2 and then regulate mRNA translation.

### Effects of miR-153 on the physiological and biochemical states of IPEC-J2 cells

**Figure 4:**
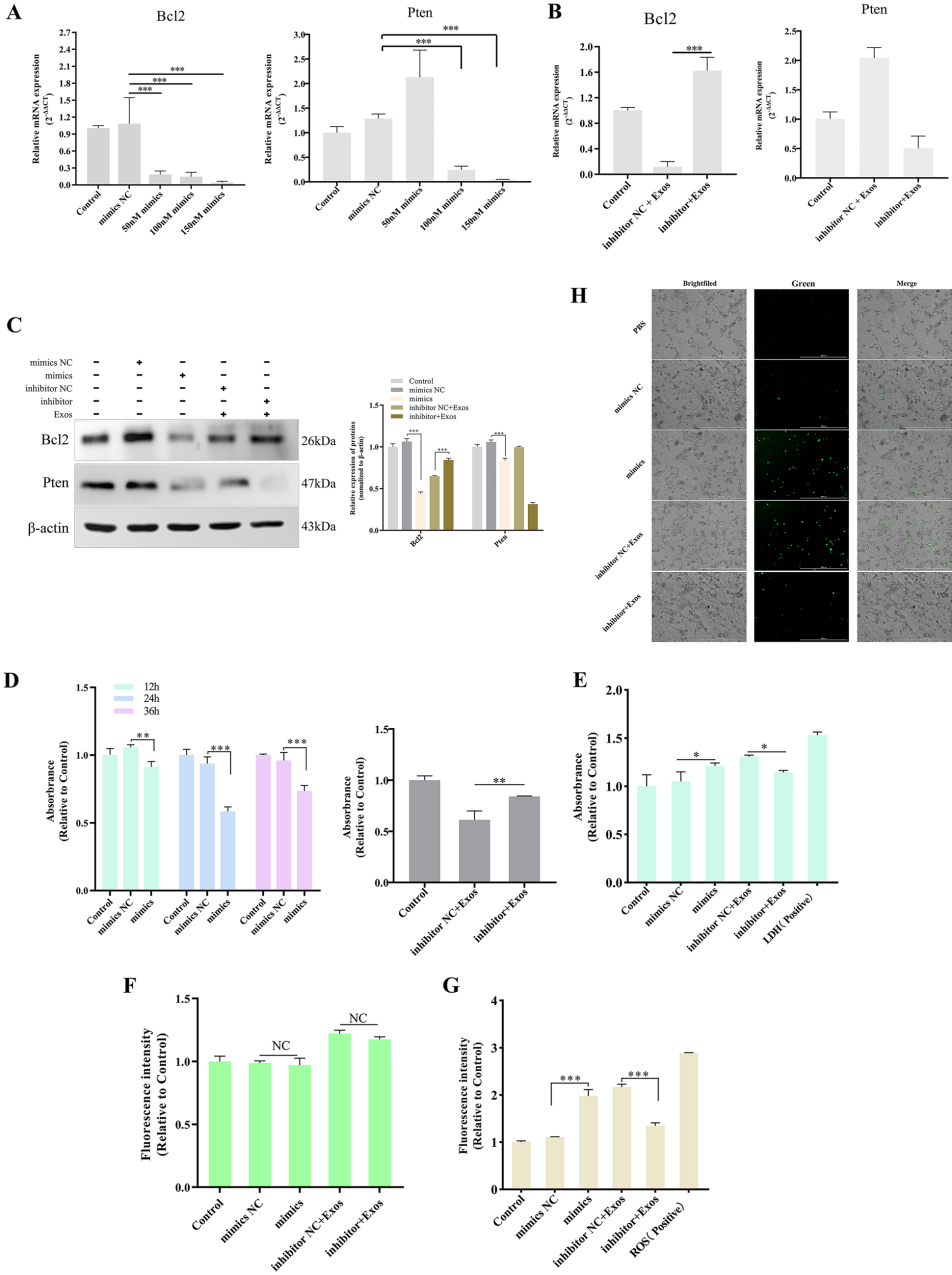
** Effects of miR-153 on IPEC-J2 cells.** IPEC-J2 cells were transfected with miR-153 mimics or transfected with miR-153 inhibitor and then incubated with *Ts*Exos. Then, the assays were carried out. **A** Determination of the transfection efficiency of different concentrations of miR-153 mimics. **B** Changes in the transcription level of target genes after inhibiting TsExo-cargoed miR-153. **C** Western blotting was performed to detect the modulation of target genes via miR-153. The effect of miR-153 on cell proliferation through the CCK-8 assay at OD_450_ values **D**, on cell damage by assessing the level of LDH at OD490 **E**, on cell permeability by detecting fluorescence intensity of FITC-dextran at 493-nm excitation and 517-nm emission wavelengths **F**, on cell oxidative stress detected by the fluorescence microplate reader at 488-nm excitation and 525-nm emission wavelengths **G**, and the fluorescence microscope at 20× PL FL magnification **H**. All assays were performed in triplicate, and data are presented as the mean ± SD. *P < 0.05, **P < 0.01, and ***P < 0.001 indicate the significance levels of the comparisons between the mimic NC group and the mimic group, as well as between the inhibitor NC group and the inhibitor group.

Bcl2 and Pten protein levels were determined to evaluate the efficiency of the transfection of the miR-153 mimics in IPEC-J2 cells. The qPCR results demonstrated that in a comparison of the PBS group (the blank control group consisted of untreated cells) with the mimic NC group, no changes were observed in the transcription levels of Bcl2 and Pten, indicating that the infection process had no impact on the IPEC-JE cells. (Note: In the subsequent results analysis, the PBS control group was compared to the miR-153 mimic NC group by default, without specific description, indicating no significant difference between the two.) Treatment with 50 nM (nmol/L) or 100 nM miR-153 mimics significantly decreased the transcriptional levels of Bcl2 or Pten (*P* < 0.001), respectively, and the levels of Bcl2 and Pten remained decreased as the concentration further increased (Figure 4A). However, the qPCR results validated the reliability of the dual luciferase reporter gene assay. For convenience, 100 nM was selected as the working concentration. To avoid the effect on target gene expression, we inhibited miR-153 activity using a miR-153 inhibitor. The levels of Bcl2 and Pten were remeasured in cells transfected with miR-153 inhibitor followed by coculture with *Ts*Exos. The results showed that the transcriptional level of Bcl2 in the miR-153 inhibitor group was significantly increased and that it rescued the low level caused by miR-153 compared with the inhibitor NC (negative control) (*P* < 0.001). However, the content of Pten remained low, indicating that the ability of miR-153 to reduce Pten expression in *Ts*Exos was impeded by other substances (Figure 4B). The results of Western blotting were consistent with those of qPCR, revealing that *Ts*Exo-packaged miR-153 only targeted Bcl2 for degradation (Figure 4C). Hence, miR-153 and its target mRNA Bcl2 were investigated in follow-up assays.

The CCK-8 kit was used to measure the effect of *Ts*Exo-packaged miR-153 on cell proliferation. At the three measurement time points (12, 24, and 36 h), the extent of cell vitality reduction was the highest at 24 h after transfection (*P* < 0.001). Therefore, 24 h post-transfection with miRNA mimics was chosen. In contrast, the miR-153 inhibitor group had a higher proliferation than the inhibitor NC group (Figure 4D). Similarly, the LDH and ROS assays revealed that the LDH and ROS contents in the miR-153-transfected cells were significantly higher than those in the control cells (*P* < 0.05; *P* < 0.001). In contrast, the miR-153 inhibitor reduced the accumulation of LDH or ROS in the IPEC-J2 cells treated with *Ts*Exos (*P* < 0.05; *P* < 0.001) (Figures 4E, G, and H). Nevertheless, in the membrane permeability assay, no significant changes were observed by assessing the FITC-dextran levels, which permeated through the monolayer to the lower chamber (Figure 4F). Taken together, these results suggested that TsExo-delivered miR-153 could reduce IPEC-J2 viability, cause cell damage, and lead to high levels of cellular oxidative stress without altering cell permeability.

### Effects of miR-153 on cell apoptosis

**Figure 5:**
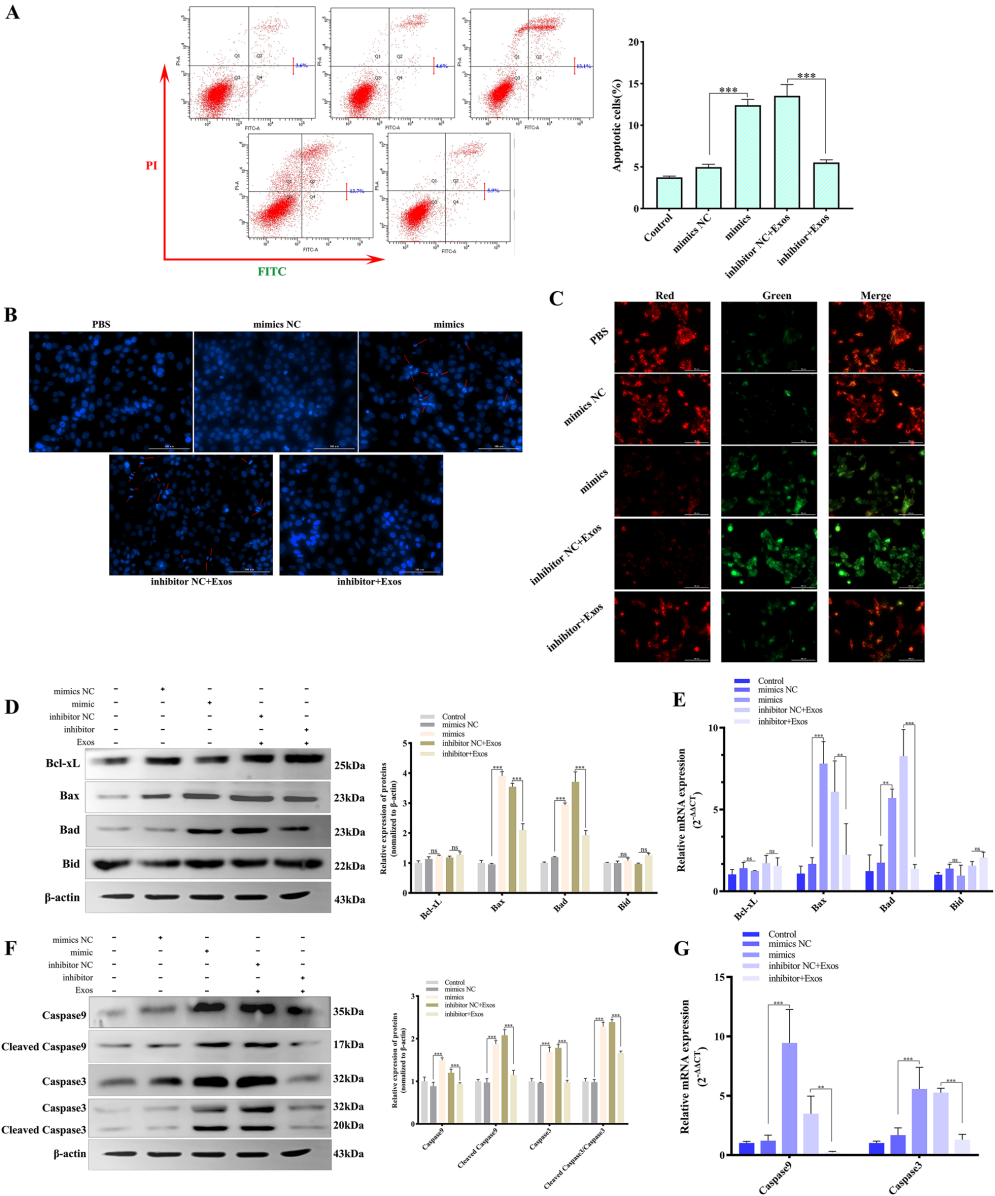
**
Effects of miR-153 on cell apoptosis.** IPEC-J2 cells were transfected with miR-153 mimics or transfected with miR-153 inhibitor and then incubated with *Ts*Exos. Then, the assays were carried out. Apoptosis of IPEC-J2 cells was determined by staining with annexin V and PI followed by flow cytometry (**A**) or by staining with Hoechst 33258 (**B**). (**C**) Detection of variations in mitochondrial membrane potential by performing JC-1 staining. Changes in Bcl2 family proteins were verified by Western blotting (**D**) and qPCR (**E**). Verification of apoptosis protein expression changes by Western blots (**F**) and qPCR (**G**). All assays were performed in triplicate, and data are presented as the mean ± SD. *P < 0.05, **P < 0.01, and ***P < 0.001 indicate the significance levels of the comparisons between the mimic NC group and the mimic group, as well as between the inhibitor NC group and the inhibitor group.

Bcl2, which is targeted by miR-153, is an antiapoptotic molecule functioning through the mitochondrial apoptotic pathway. As a result, we speculated that miR-153 affected the mitochondria-mediated endogenous apoptosis pathway. For the cell apoptosis assays, the FCM results showed that compared with the NC group, the miR-153 mimic group had a 2.85-fold increase in the apoptosis rate, and the miR-153 inhibitor group had a 2.32-fold decrease in the apoptosis rate compared with the inhibitor NC group (Figure 5A). As shown in Figure 5B, the nuclei of live cells stained with Hoechst 33,258 were homogenously stained blue, whereas the miR-153 mimic group had more apoptotic cells with nuclear condensation and deep staining. In contrast, the number of deeply stained apoptotic cells in the miR-153 inhibitor group was significantly decreased compared with that in the inhibitor NC group. For the JC-1 assay, the normal cells in the PBS and NC groups held polarized mitochondrial membrane potentials and exhibited red fluorescence owing to the accumulation of JC-1 in the mitochondria. However, as the miR-153 content increased, the depolarized mitochondrial membrane potential decreased in JC-1 red fluorescence and increased in JC-1 green fluorescence, indicating that the mitochondrial membrane potential was hampered. Conversely, cells exhibited red fluorescence, and the number of green fluorescent cells was reduced by inhibiting miR-153 function (Figure 5C).

Directly thereafter, the members of the Bcl2 family and apoptotic proteins were detected. The results of qPCR and Western blotting displayed higher Bax and Bad expression in the miR-153 mimic group than in the NC group (*P* < 0.001). In contrast, a decrease in miR-153 accumulation lowered the content of Bax and Bad. Furthermore, the results of qPCR and Western blotting demonstrated that miR-153 had no impact on Bcl-xL and Bid expression, respectively (Figures 5D and E). In addition, the apoptosis executioner proteins Caspase 9 and Caspase 3 were markedly increased after an increase in exogenous miR-153 levels in IPEC-J2 cells; in contrast, the levels of these proteins were reduced by the miR-153 inhibitor (Figures 5F and G). In summary, the results suggested that *Ts*Exo-cargoed miR-153 could induce an increase in apoptosis, possibly by targeting Bcl2 and/or increasing Bax and Bad levels to affect mitochondria-mediated apoptosis and subsequently activating Caspase 9 and Caspase 3 to execute the apoptotic process.

### Effects of miR-153 on apoptosis-related pathways

**Figure 6:**
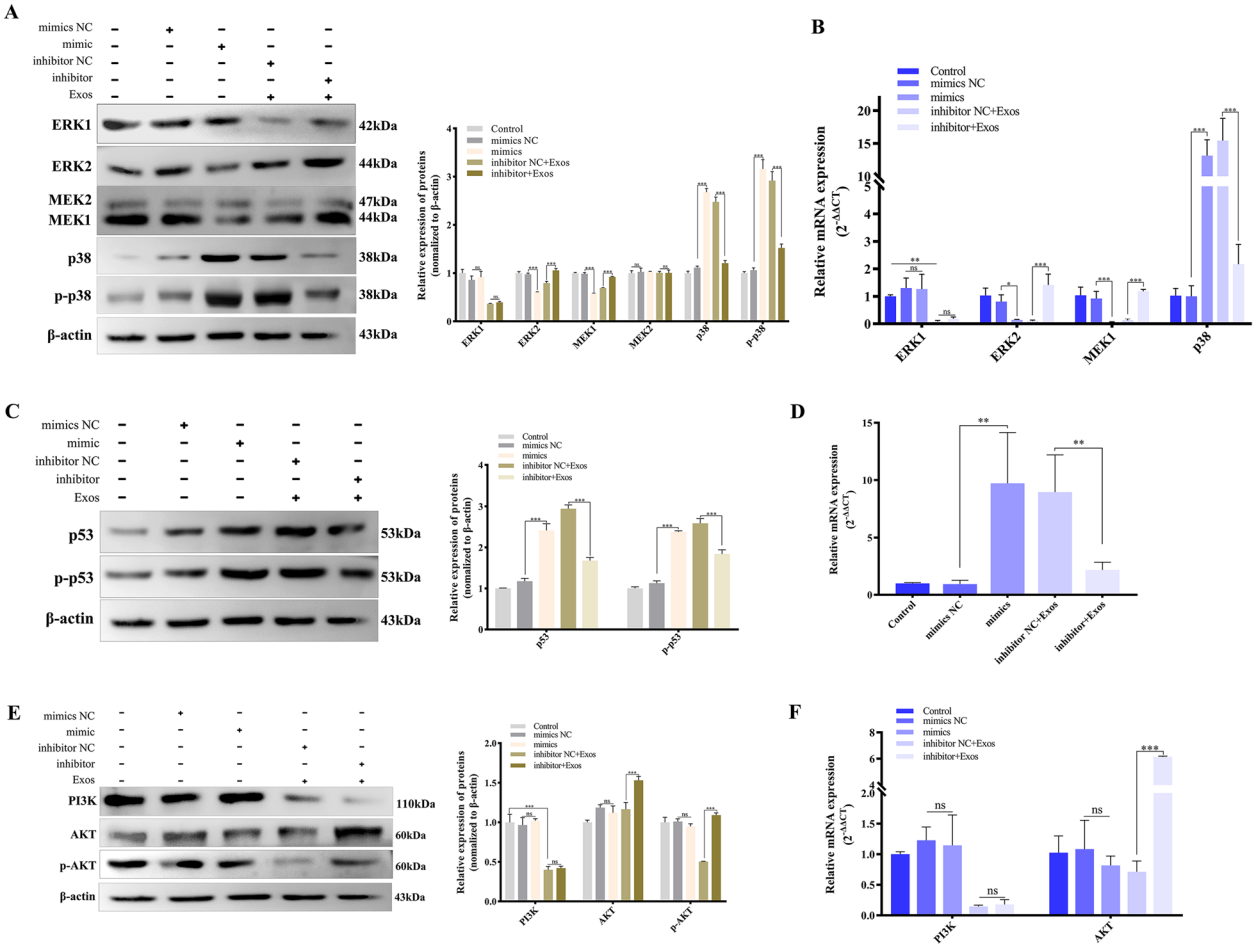
**
Effects of miR-153 on apoptosis-regulated signalling pathways in IPEC-J2 cells.** IPEC-J2 cells were transfected with miR-153 mimics or transfected with miR-153 inhibitor and then incubated with *Ts*Exos. Then, the assays were carried out. The protein expression levels of key effectors in the MAPK, p53, and PI3K/AKT signalling pathways were evaluated using Western blotting (**A**,** C**,** E**) and qPCR (**B**,** D**,** F**). All assays were performed in triplicate, and data are presented as the mean ± SD. *P < 0.05, **P < 0.01, and ***P < 0.001 indicate the significance levels of the comparisons between the mimic NC group and the mimic group, as well as between the inhibitor NC group and the inhibitor group.

To evaluate the effects of TsExo-delivered miR-153 on the apoptosis-regulated signalling pathways in IPEC-J2 cells, we determined the transcriptional and protein levels of molecules involved in the MAPK, p53, and PI3K/AKT signalling pathways. Both qPCR and Western blotting revealed that genes related to the ERK-p38-MAPK signalling pathways were involved and that TsExo-packaged miR-153 could significantly reduce ERK2 and MER1 expression and significantly increase p38 and phosphorylated p38 (p-p38) levels. Moreover, the miR-153 mimics did not affect ERK1 and MEK2 protein expression in IPEC-J2 cells; however, *Ts*Exos could decrease the expression of ERK1 without any clear reason (Figures 6A and B). Similarly, analysis of the p53 signalling pathway showed that miR-153 within *Ts*Exos could decrease protein p53 and phosphorylated p53 (p-p53) levels (Figures 6C and D). Furthermore, the findings revealed that for the PI3K/AKT signalling pathway, *Ts*Exos, rather than miR-153, significantly decreased the expression level of PI3K while significantly increasing the expression levels of AKT and phosphorylated AKT (p-AKT) (Figures 6E and F).

In summary, miR-153 derived from *Ts*Exos could promote cell apoptosis by regulating the ERK-p38-MAPK and p53 signalling pathways instead of the PI3K/AKT signalling pathway, specifically by reducing the expression levels of ERK2 and MEK1 and increasing those of p38 and p53.

## Discussion

Exosomes produced by parasites are considered “bridges” that facilitate communication between parasites and their hosts during infection, serving as an additional means by which helminths manipulate their hosts [20]. In a previous study, it was found that exosomes derived from *T. spiralis* could affect the barrier function and immune responses of intestinal epithelial cells. In general, exosomes are small endocytic membrane vesicles that are released by many cells, mediating cell‒cell communication by delivering their cargoes, such as miRNAs [13, 21]. The exosomal shuttle miRNA can be functional in this new location, modifying recipient cell protein production and gene expression and in turn providing the necessary signals to modulate recipient cell functions [22]. It is important to identify the miRNAs within *Ts*Exos that interact with the host immune pathway to enhance our understanding of the interplay between the parasite and its host. In the present study, we aimed to discuss the effects of TsExo-delivered miR-153 on IPEC-J2 cells.

miRNA can regulate the transcription and translation of mRNA by binding to the 3′-UTR of the target mRNA, leading to mRNA cleavage or translational repression. Therefore, the function of the miRNA is determined by its downstream target gene [23]. In addition, in the mechanism of different diseases, the same miRNA can regulate multiple target genes and serve different functions. A study reported that miR-153 regulates the apoptosis and autophagy of cardiomyocytes by targeting Mcl-1 [24]. Other studies have reported that miR-153 promotes breast cancer cell apoptosis by targeting HECTD3 and suppresses human laryngeal squamous cell carcinoma migration by targeting the SNAI1 gene [25, 26]. In summary, numerous studies have demonstrated that miR-153 plays a critical role in the regulation of tumours, cancer and neurological diseases [26, 27]. Understanding miRNA function and how it regulates biological processes depends on elucidating the miRNA targets. In this study, the targeting relationship between the candidate genes Agap2, Bcl2, Pten, and miR-153 was initially validated. The results indicated that Bcl2 and Pten were the downstream target genes of miR-153. Nevertheless, the functions of miR-153 within *Ts*Exos were limited by unknown components that repress the translation of its own target Pten. Thus, we focused on elucidating the functions of miR-153 given the functions of the target gene Bcl2.

Some studies have suggested that miR-153 participates in and regulates multiple cellular biological processes, including cell proliferation, oxidative stress and cell permeability [28–30]. TsExo-delivered miR-153 decreased the viability of IPEC-J2 cells, induced cellular damage, and increased oxidative stress levels, while not affecting cell permeability, demonstrating that miR-153 played a vital role in modulating multiple cellular biological processes affected by *Ts*Exos.

The Bcl2 family of proteins is considered the main intracellular regulator of the apoptotic process and is involved in the mitochondria-dependent apoptosis pathway. Cellular survival controlled by the apoptotic process is determined by the equilibrium between the upregulation and downregulation of proapoptotic proteins such as Bax, Bid, and Bad and antiapoptotic proteins such as Bcl-xL [31, 32]. Moreover, the target gene Bcl2 is one of the products of the Bcl2 family and exhibits antiapoptotic and prosurvival functions [33]. In the present study, we demonstrated that miR-153 is a proapoptotic factor in IPEC-J2 cells, consistent with the findings of other studies that miR-153 functions as an apoptosis-inducing factor [34, 35]. MiR-153 could influence the process of IPEC-J2 cell apoptosis by increasing the expression of Bax and Bad associated with the mitochondrial-mediated apoptotic pathway. However, further studies are warranted to determine whether the downregulation of the target gene Bcl2 mediated by miR-153 enhances the expression of Bax and Bad and ultimately leads to apoptosis. Notably, apoptosis is a vital protective mechanism in organisms and plays a crucial role in maintaining host intestinal barrier function. However, the apoptosis induced by parasites is not always beneficial to the host; instead, it serves as an adaptive strategy employed by parasites. By manipulating host cell apoptosis, parasites can evade detection and clearance by the host immune system [36]. In this study, the induction of intestinal epithelial cell apoptosis by miR-153 contained in *T*sExos may be attributed to this mechanism.

The MAPK signalling pathway regulates several biological processes through various cellular mechanisms, including apoptosis. The MAPK family includes the ERK, JNK, and p38 MAPK subfamilies. After the MAPK signalling cascade is activated, ERK functions as an antiapoptotic pathway by its key proteins ERK and MEK, whereas p38 exerts proapoptotic effects during apoptosis [37, 38]. Furthermore, the p53 pathway is a classic apoptosis pathway. The key gene p53 is an activator of cell apoptosis, and inhibiting the p53 gene significantly reduces cell apoptosis [39]. In addition, the PI3K/Akt-mediated signalling pathway plays a prosurvival role against apoptosis [40]. Many studies have emphasized the importance of miR-153 in regulating apoptosis-related signalling pathways. For example, Yang et al. found that miR-153 overexpression increases phosphorylated p38 and ATF2 and downstream differentiation markers in GSCs [41]. Another study showed that Krüppel-like factor 5 (KLF5) is a target of miR-153 and can interact with p53 [42]. In the present study, it was concluded that cellular apoptosis was triggered by miR-153 and achieved by regulating the ERK-p38-MAPK and p53 signalling pathways, as opposed to the PI3K/AKT signalling pathway. Moreover, many studies have reported that Bcl2, as a crucial intersection, highlights its involvement in a wide web of apoptosis-regulated signalling pathways, including the MAPK, p53 and PI3K/AKT signalling pathways [43–45]. However, whether miR-153 modulates the MAPK and p53 signalling pathways by targeting Bcl2 remains unknown.

In conclusion, miR-153 delivered by *Ts*Exos participated in the regulation of various biological processes in IPEC-J2 cells by inhibiting the target gene Bcl2. MiR-153 could reduce cell viability and cause cell damage and excessive oxidative stress. Furthermore, it was confirmed that *Ts*Exo-cargoed miR-153 could trigger apoptosis, possibly by repressing Bcl2 and/or increasing Bax and Bad levels to affect mitochondria-mediated apoptosis or by regulating the apoptosis-related signalling pathways ERK-p38-MAPK and p53. Therefore, miR-153 within *Ts*Exos might be a key factor in the interaction between *T. spiralis* and host cells.

## Supplementary Information

**Additional file 1. Sequencing comparisonresults of WT-Agap2, Bcl2 and Pten**.“NCBI WT-Agap2 3'-UTR”represents the Agap2 3′-UTR sequences obtainedfrom GenBank. “Sequencing WT-Agap2 3′-UTR” represents the sequencing results of the WT-Agap2 3′-UTR. Additionally,Bcl2 and Pten are similar to Agap2.

**Additional file 2. The 3′-UTR point mutationsequences**.The 3′-UTR point mutation sequences for Agap2, Bcl2and Pten. Point mutations were introduced by asingle base substitution.

**Additional file 3. Sequencing comparisonresults of MUT-Agap2, Bcl2 and Pten**.“Designed MUT-Agap23′-UTR” represents the mutation sequences of the Agap23'-UTR. “Sequencing MUT-Agap2 3′-UTR” represents the sequencing results ofMUT-Agap2 3′-UTR. Additionally, Bcl2 and Ptenare similar to Agap2.

**Additional file 4. Full names for the gene abbreviation.**

## Data Availability

The datasets used or analysed during the current study are available from the corresponding author on reasonable request.
