# Supplementary figures and images for: Exosome-delivered miR-153 from Trichinella spiralis promotes apoptosis of intestinal epithelial cells by downregulating Bcl2
Source: Vet Res. 2023 Jun 28;54:52. doi: 10.1186/s13567-023-01186-6 (PMC10304724; doi:10.1186/s13567-023-01186-6)

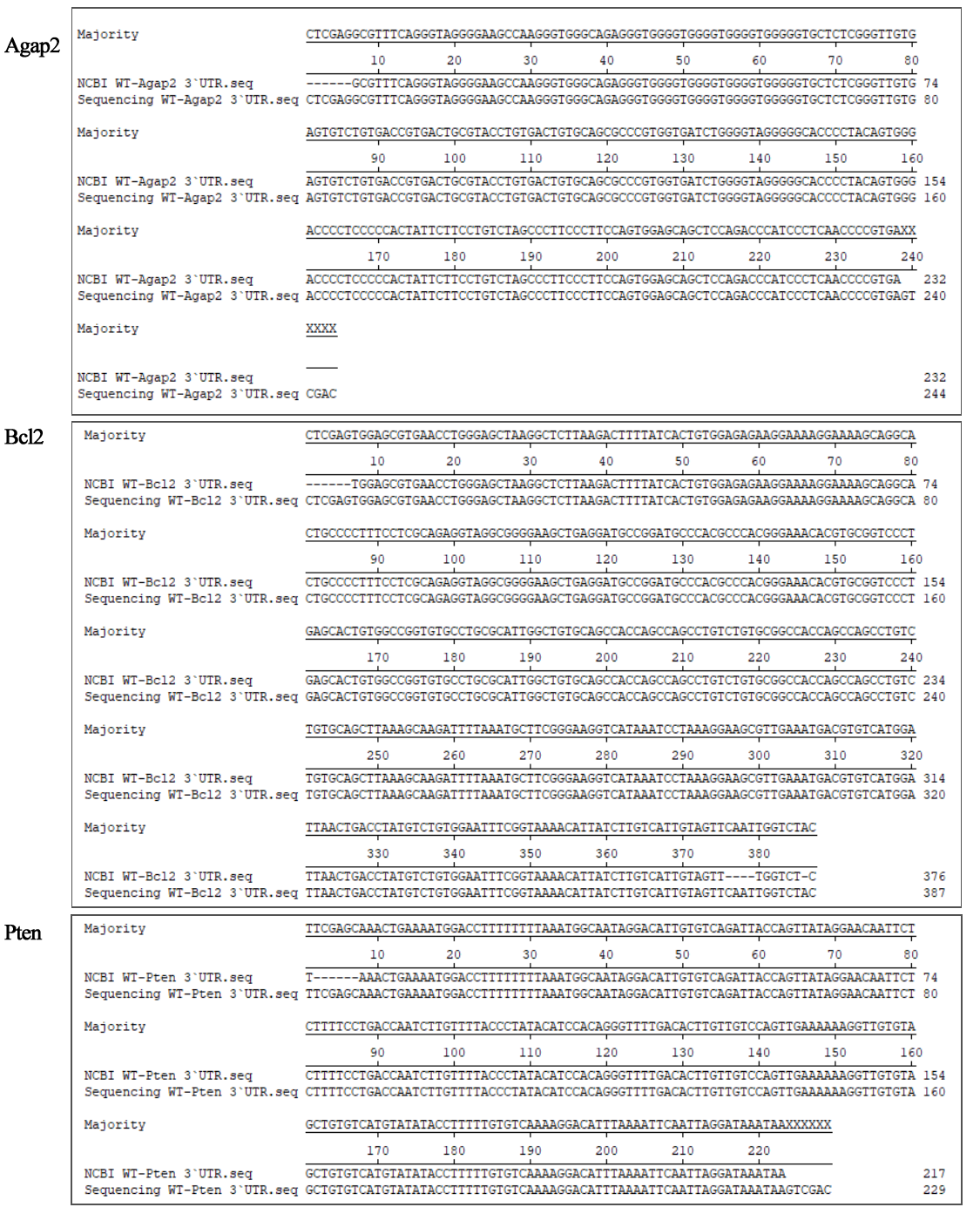

Supplement: Supplementary file 1 — Additional file 1. Sequencing comparisonresults of WT-Agap2, Bcl2 and Pten.“NCBI WT-Agap2 3'-UTR”represents the Agap2 3′-UTR sequences obtainedfrom GenBank. “Sequencing WT-Agap2 3′-UTR” represents the sequencing results of the WT-Agap2 3′-UTR. Additionally,Bcl2 and Pten are similar to Agap2. [file 13567_2023_1186_MOESM1_ESM.png]

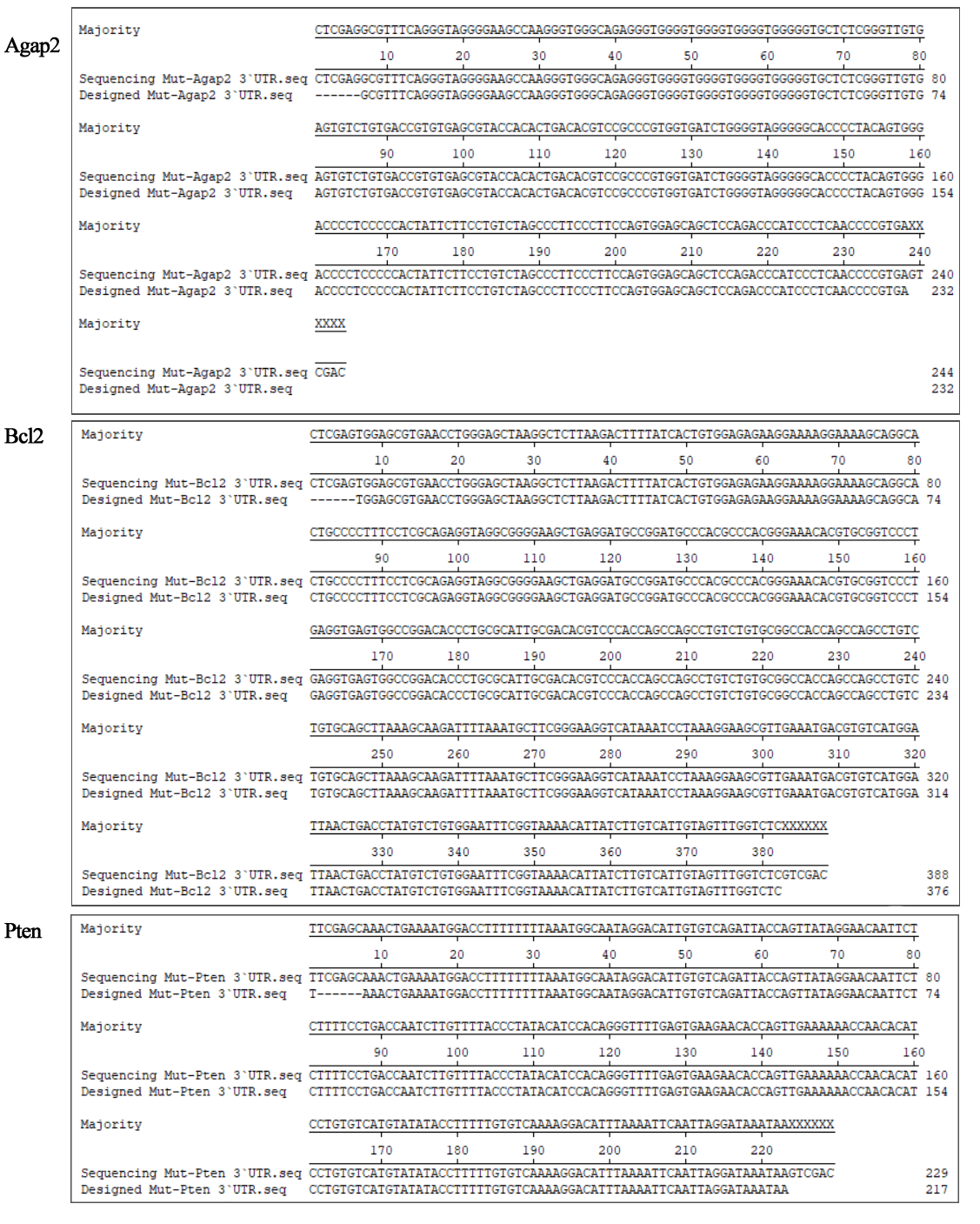

Supplement: Supplementary file 3 — Additional file 3. Sequencing comparisonresults of MUT-Agap2, Bcl2 and Pten.“Designed MUT-Agap23′-UTR” represents the mutation sequences of the Agap23'-UTR. “Sequencing MUT-Agap2 3′-UTR” represents the sequencing results ofMUT-Agap2 3′-UTR. Additionally, Bcl2 and Ptenare similar to Agap2. [file 13567_2023_1186_MOESM3_ESM.png]
