# Supplementary material for: Exosome-delivered miR-153 from Trichinella spiralis promotes apoptosis of intestinal epithelial cells by downregulating Bcl2
Source: Vet Res. 2023 Jun 28;54:52. doi: 10.1186/s13567-023-01186-6 (PMC10304724; doi:10.1186/s13567-023-01186-6)
Supplement: Supplementary file 2 — Additional file 2. The 3′-UTR point mutationsequences.The 3′-UTR point mutation sequences for Agap2, Bcl2and Pten. Point mutations were introduced by asingle base substitution. [file 13567_2023_1186_MOESM2_ESM.docx]

The 3’UTR point mutation sequences for Agap2 (232 bp):

gcgtttcagggtaggggaagccaagggtgggcagagggtggggtggggtggggtgggggtgctctcgggttgtgagtgtctgtgaccgtgTGAgcgtaccACACTGACACGTCcgcccgtggtgatctggggtagggggcacccctacagtgggacccctcccccactattcttcctgtctagcccttcccttccagtggagcagctccagacccatccctcaaccccgtga

The 3’UTR point mutation sequences for Bcl2 (376 bp):

tggagcgtgaacctgggagctaaggctcttaagacttttatcactgtggagagaaggaaaaggaaaagcaggcactgcccctttcctcgcagaggtaggcggggaagctgaggatgccggatgcccacgcccacgggaaacacgtgcggtccctgagGTGAgtggccggACACcctgcgcattgCGACACGTCccaccagccagcctgtctgtgcggccaccagccagcctgtctgtgcagcttaaagcaagattttaaatgcttcgggaaggtcataaatcctaaaggaagcgttgaaatgacgtgtcatggattaactgacctatgtctgtggaatttcggtaaaacattatcttgtcattgtagtttggtctc

The 3’UTR point mutation sequences for Pten (217 bp):

taaactgaaaatggaccttttttttaaatggcaataggacattgtgtcagattaccagttataggaacaattctcttttcctgaccaatcttgttttaccctatacatccacagggttttgaGTGAAgAACAccagttgaaaaaaCCAACACATCctgtgtcatgtatatacctttttgtgtcaaaaggacatttaaaattcaattaggataaataa

Note: Capital letters represent point mutation sequences.
