# Supplementary material for: Exosome-delivered miR-153 from Trichinella spiralis promotes apoptosis of intestinal epithelial cells by downregulating Bcl2
Source: Vet Res. 2023 Jun 28;54:52. doi: 10.1186/s13567-023-01186-6 (PMC10304724; doi:10.1186/s13567-023-01186-6)
Supplement: Supplementary file 4 — Additional file 4. Full names for the gene abbreviation. [file 13567_2023_1186_MOESM4_ESM.docx]

**Full names for the gene abbreviation**

Agap2: ArfGAP with GTPase domain, ankyrin repeat and PH domain 2;

Bcl2: B-cell lymphoma 2;

Pten: phosphatase and tensin homolog;

Bcl-xL: apoptosis regulator Bcl-X;

Bax: Bcl-2-associated X protein;

BAD: Bcl-2-associated agonist of cell death;

BID: BH3 interacting domain death agonist;

ERK: Extracellular signal-regulated kinase;

MEK: Mitogen-activated protein kinase kinase;

p38: p38 mitogen-activated protein kinase;

p53: tumor protein p53

PI3K: Phosphatidylinositol 3-kinase;

AKT: AKT serine/threonine kinase;

GAPDH: Glyceraldehyde-3-phosphate dehydrogenase.
